# Human rpL3 plays a crucial role in cell response to nucleolar stress induced by 5-FU and L-OHP

**DOI:** 10.18632/oncotarget.2591

**Published:** 2014-11-28

**Authors:** Davide Esposito, Elvira Crescenzi, Vinay Sagar, Fabrizio Loreni, Annapina Russo, Giulia Russo

**Affiliations:** ^1^ Department of Molecular Medicine and Medical Biotechnology, University of Naples “Federico II”, Naples 80131, Italy; ^2^ Department of Oncological Sciences, Icahn School of Medicine at Mount Sinai, New York, NY, USA; ^3^ Institute of Experimental Endocrinology and Oncology-CNR, Naples 80131, Italy; ^4^ Department of Biology, University of Rome ‘Tor Vergata’, Rome 00133, Italy; ^5^ Department of Pharmacy, University of Naples “Federico II”, Naples 80131, Italy

**Keywords:** p21, ribosomal protein, 5-FU, Oxaliplatin, DNA repair

## Abstract

Recent evidence showed that a variety of DNA damaging agents including 5-FU and L-OHP impairs ribosomal biogenesis activating a ribosomal stress pathway. Here, we demonstrate that in lung and colon cancer cell lines devoid of p53, the efficacy of 5-FU and L-OHP chemotherapy depends on rpL3 status. Specifically, we demonstrate that ribosomal stress induced by 5-FU and L-OHP is associated to up-regulation of rpL3 and its accumulation as ribosome-free form. We show that rpL3 participates in the cell response to chemotherapy acting as a critical regulator of cell cycle, apoptosis and DNA repair, by modulating p21 expression. Moreover, we demonstrate that rpL3 is able to control DNA repair also independently from p21 status of cell. It is noteworthy that silencing of rpL3 abolishes the cytotoxic effects of 5-FU and L-OH indicating that the loss of rpL3 makes chemotherapy drugs ineffective. Taking together our results shed light on 5-FU and L-OHP mechanism of action and contribute to more effective clinical use of these drugs in cancer therapy.

## INTRODUCTION

A major role of nucleolus is the production of small and large ribosome subunits; however as involved in additional cellular functions, provides a link between ribosome subunits biosynthesis, cell cycle progression and stress signaling [1–3]. Recently, some evidence, indicate that 5-fluouracil (5-FU) and Oxaliplatin (L-OHP) represent two chemotherapeutic molecules exerting inhibitory effect on ribosome biogenesis [4]. Three different levels for perturbation of ribosome biogenesis by chemotherapeutic drugs have been defined, i.e. inhibition of rRNA gene transcription, inhibition of early and late maturation of rRNA precursors, and perturbation linked to disintegration of nucleolar structures [5–7]. Drugs which inhibit the rRNA transcription and early processing of 32S rRNA precursor, such as L-OHP, promote the nucleoplasmic translocation of nucleophosmin (NPM), a well-known marker of nucleolar disruption [8]. However, nucleolar breakdown does not seem to be an essential step for activation of the ribosomal stress pathway. In fact, 5-FU, which inhibits rRNA late processing steps, does not induce NPM translocation into the nucleoplasm.

5-FU and L-OHP are widely used for therapy of a variety of solid tumors. Increased understanding of the mechanism of action of these two drugs has led to the development of strategies that enhance their anticancer activity. Despite these advances, response rates to 5-FU-based chemotherapy, alone or in combination with L-OHP, does not exceed 40–50%. In addition, development of drug resistance during the therapy remains a major problem [9].

During the last few years, the effects of 5-FU on cell growth arrest and apoptosis have been attributed to a p53-dependent stress signaling pathway, activated in response to disruption of ribosomal biogenesis [10]. This type of p53-activating stimulus involves a subset of ribosomal proteins (rp), including rpL11, rpL23, rpL5, rpS7 [11]. These proteins affect human double minute-2 (HDM2)-p53 circuit and lead to accumulation of p53 causing p53-dependent cell-cycle arrest [12–14].

It is known that most cancer cells contain mutant p53 or no p53 at all [15, 16]. In recent years, some data revealed the existence of p53-independent mechanisms involving ribosomal proteins that link nucleolar stress to cell cycle arrest [17–19].

We have studied post-transcriptional regulatory strategies of mammalian ribosomal proteins (r-proteins), and we demonstrated that human rpL3 autoregulates its own expression through alternative splicing associate to nonsense-mediated mRNA decay. We have also identified hnRNP H1, NPM and KHSRP as transacting factors involved in the regulation of the alternative splicing of rpL3 gene and analysed their role in rpL3 autoregultory circuit [20].

We have recently demonstrated that human rpL3 exerts an extra-ribosomal function resulting in the induction of p21-dependent cell growth arrest and apoptosis in the absence of p53 [21]. In this paper, we demonstrate that ribosome-free rpL3 plays a critical role in cell response to ribosome stress induced by 5-FU and L-OHP treatments. Our data show that rpL3 is involved in drug-induced cell-cycle arrest, apoptosis and DNA repair by controlling p21 expression. In addition, rpL3 is also able to control DNA repair in a p21-independent manner. Finally, we report that the absence of rpL3 is dramatically associated to ineffectiveness of 5-FU and L-OHP.

## RESULTS

### 5-FU and L-OHP treatments increase the ribosome-free form of rpL3

**Figure 1:**
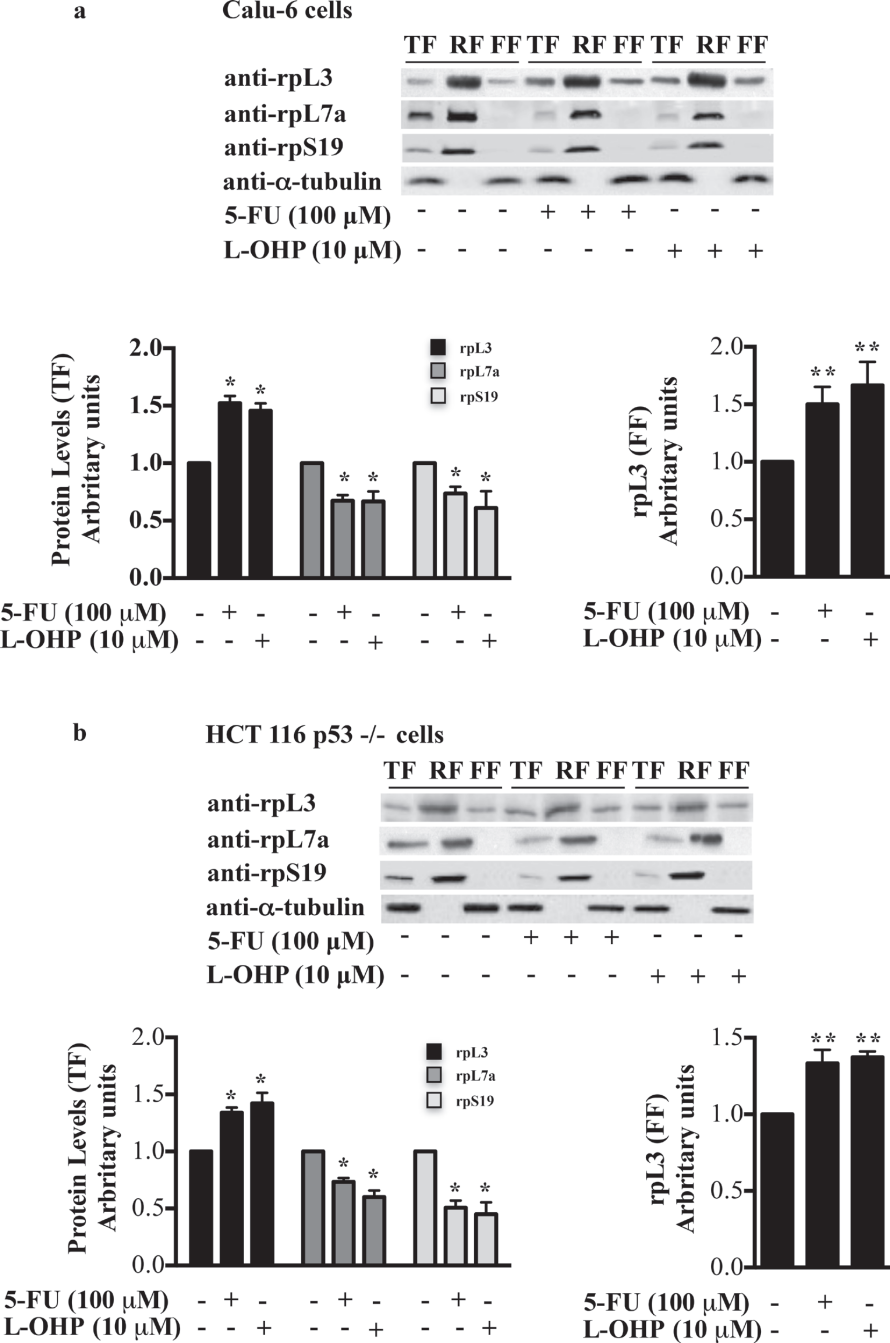
Distribution profile of rpL3 protein upon drug treatments **(a)** Calu-6 cells, untreated and treated with 100 μM 5-FU or 10 μM L-OHP for 24 h, were collected, lysed (total fraction, TF) and fractionated to obtained the ribosome-associated fraction (RF) and ribosome-free fractions (FF). The fractions were analyzed by western blotting with the indicated antibodies. The per-cell ratio of the amount of protein loaded onto a gel was TF:RF:FF/1:10:1. Quantification of rpL3, rpL7a and rpS19 in TF (panel on the left) and quantification of rpL3 in FF (panel on the right) are shown. **(b)** Results from analogous experiments performed in HCT 116 p53−/− cells are shown. Results illustrated in Figures 1–8 are representative of three independently performed experiments.

Ribosome biogenesis represents a target for a large variety of drugs for cancer therapy [3, 22]. It is known that upon drug-induced ribosomal stress some r-proteins accumulate as ribosome-free forms and induce cell growth arrest and apoptosis [14, 23–25]. To understand whether drug-induced ribosomal stress caused the accumulation of ribosome-free rpL3, we treated Calu-6 and HCT 116 p53−/− cells with 100 μM of 5-FU or 10 μM of L-OHP corresponding to the concentrations for the complete inhibition of rRNA late processing steps or rRNA gene transcription, respectively [4]. 24 h after treatments, cells were collected, lysed (total fraction, TF) and fractionated to obtain the ribosome-associated fraction (RF) and the ribosome-free fraction (FF) [19]. Proteins extracted from each fraction were analyzed by western blotting. Figures 1a and 1b show that 5-FU and L-OHP treatments caused an increase of total rpL3 level and a significant accumulation of ribosome-free rpL3. 5-FU or L-OHP failed to increase the levels of rpL7a and rpS19, two arbitrary proteins of large and small subunit respectively, which remained largely associated to the ribosome (Figures 1a and 1b).

### rpL3 is involved in the cell response to 5-FU and L-OHP

**Figure 2:**
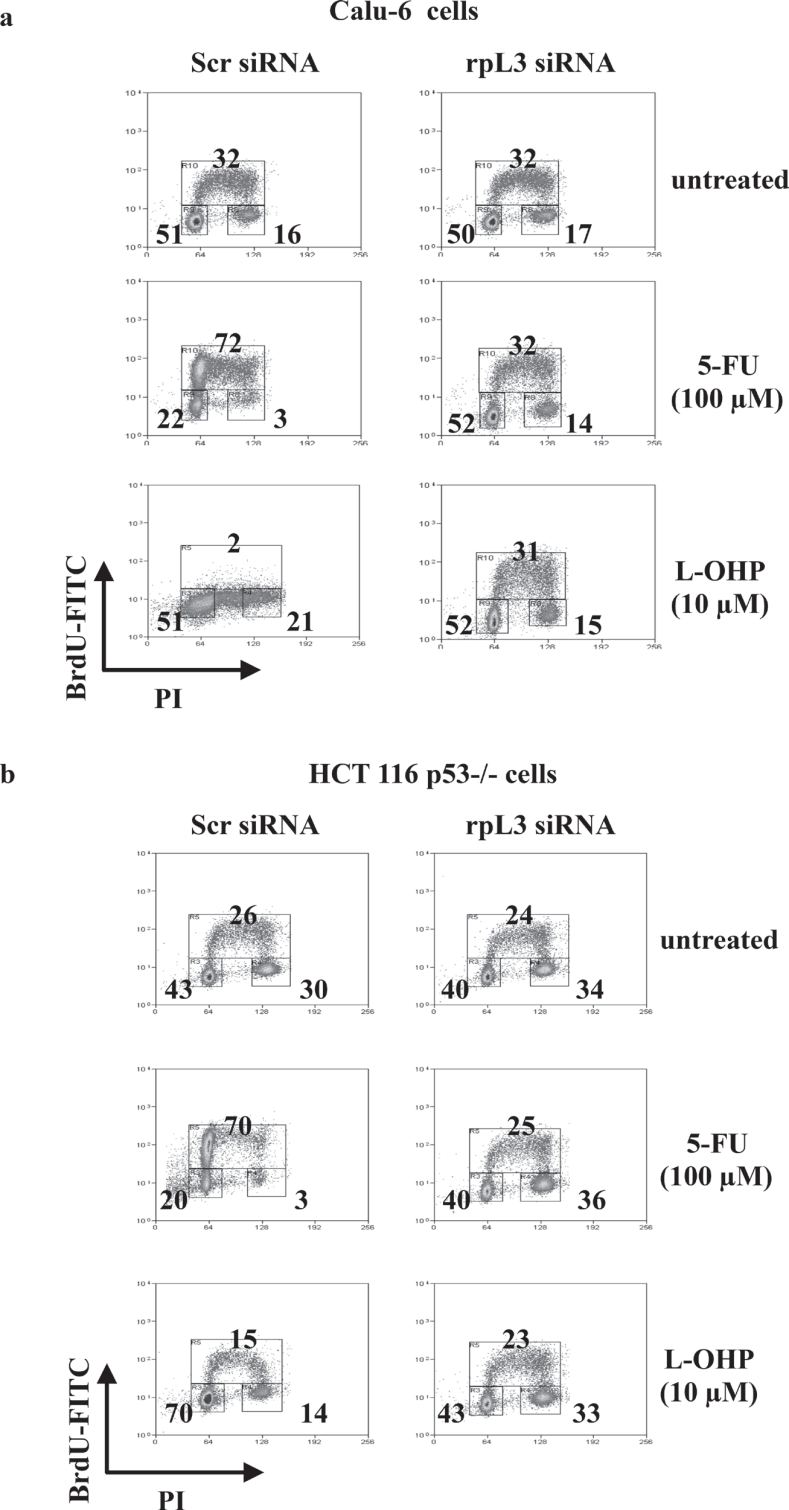
Effect of rpL3 silencing on cell cycle upon 5-FU and L-OHP treatments **(a)** Calu-6 cells and **(b)** HCT 116 p53−/− cells were transiently transfected with siRNA specific for rpL3 or scrambled siRNA (Scr) and treated with 5-FU or L-OHP for 24 h or untreated. Cells were stained with FITC conjugated anti-5-bromodeoxyuridine antibodies and counterstained with propidium iodide to analyze DNA synthesis and DNA content respectively by flow cytometry. The percentage of cells in different phases of cell cycle is shown.

In order to understand whether the ribosome-free rpL3 could be involved in the cell response to 5-FU and L-OHP, we analyzed the influence of rpL3 on drug-induced cell cycle modifications and apoptosis. To this aim, we impaired rpL3 function by incubating Calu-6 and HCT 116 p53−/− cells with siRNA specific for rpL3. Then, cells were treated with 100 μM of 5-FU or 10 μM of L-OHP. 24 h later, rpL3 protein levels were detected by western blotting (Supplementary Figure 1) and cell cycle was analyzed by flow cytometry. The tested drugs exerted different effects on the cell cycle. In the presence of rpL3 (scrambled siRNA), the addition of 100 μM of 5-FU caused S phase arrest, whereas incubation with 10 μM of L-OHP completely prevented cells to undergo DNA synthesis. Of interest, the depletion of rpL3 (rpL3 siRNA) restored normal cell cycle distribution in 5-FU or L-OHP-treated cells. Note that in untreated cells, the silencing of rpL3 did not affect the cell cycle (Figures 2a and 2b).

Analogous experiments were performed by using low doses of ActD. To this purpose, Calu-6 cells were transiently transfected with rpL3 siRNA or scrambled siRNA. Then, cells were treated with ActD 5 nM for 24 h and cell cycle was analyzed by flow cytometry. As shown in Supplementary Figure 2 the depletion of rpL3 (rpL3 siRNA) restored normal cell cycle distribution in ActD-treated cells.

**Figure 3:**
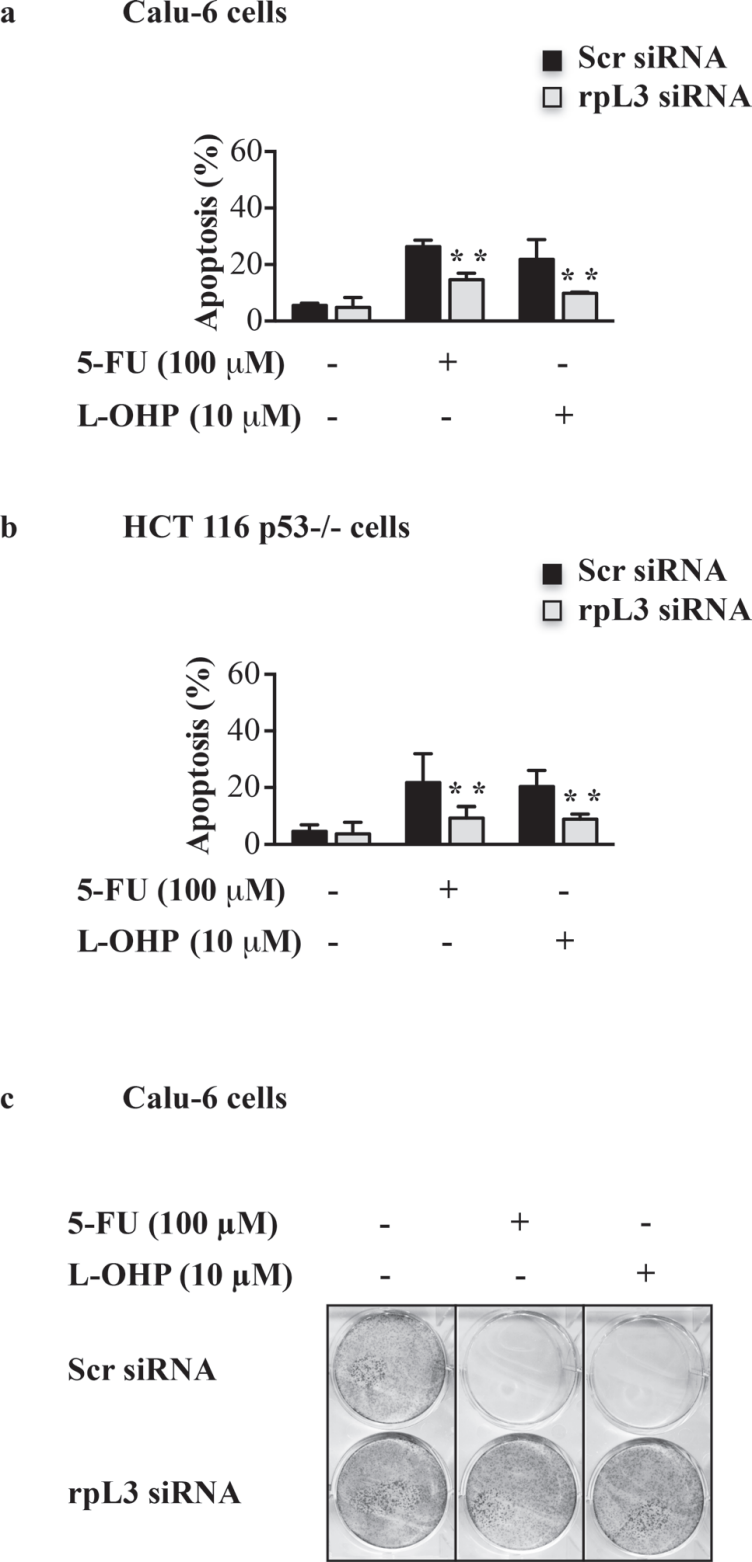
Effect of rpL3 silencing on apoptosis and cell survival upon 5-FU and L-OHP treatments **(a)** Calu-6 cells and **(b)** HCT 116 p53−/− cells were transiently transfected with siRNA specific for rpL3 or scrambled siRNA (Scr) and treated with 5-FU or L-OHP for 24 h or untreated. Then cells were analyzed for mitochondrial membrane potential by TMRE staining. Fluorescence was measured by flow cytometry. **(c)** Clonogenic assay. Calu-6 cells transiently transfected with siRNA for rpL3 or scrambled siRNA (Scr) were treated with 5-FU or L-OHP for 24 h or untreated. After 10 days, colonies were stained with methylene blue, counted and photographed.

To study the role of ribosome-free rpL3 in the apoptotic response to 5-FU and L-OHP, modifications of mitochondrial inner membrane potential (ΔΨm) were estimated by tetramethylrhodamine ethyl ester (TMRE) staining [26]. Calu-6 and HCT 116 p53−/− cells were transiently transfected with rpL3 siRNA or scrambled siRNA. Then, cells were treated with 100 μM of 5-FU or 10 μM of L-OHP for 24 h TMRE-stained cells were analyzed by flow cytometry. As expected, the percentage of apoptosis increased after 5-FU or L-OHP treatments compared to control in each cell lines (Figures 3a and 3b). Interestingly, rpL3 silencing resulted in a decrease of apoptotic cells following 5-FU or L-OHP treatments (Figures 3a and 3b).

These data suggest that rpL3 was needed to mediate 5-FU and L-OHP apoptotic cell response.

Next, we performed a clonogenic assay. To this aim, Calu-6 cells were transiently transfected with rpL3 siRNA or scrambled siRNA and treated with 100 μM of 5-FU or 10 μM of L-OHP for 24 h. In cells transfected with scrambled siRNA, the colony number was strongly reduced upon exposure to 5-FU or L-OHP thus confirming the ability of the drugs to inhibit clonogenicity. It is noteworthy that following rpL3 depletion the capacity of cells to produce colonies upon 5-FU and L-OHP treatments was comparable to the capacity of untreated cells transfected with scrambled siRNA (Figure 3c).

### rpL3 is involved in DNA damage induction

**Figure 4:**
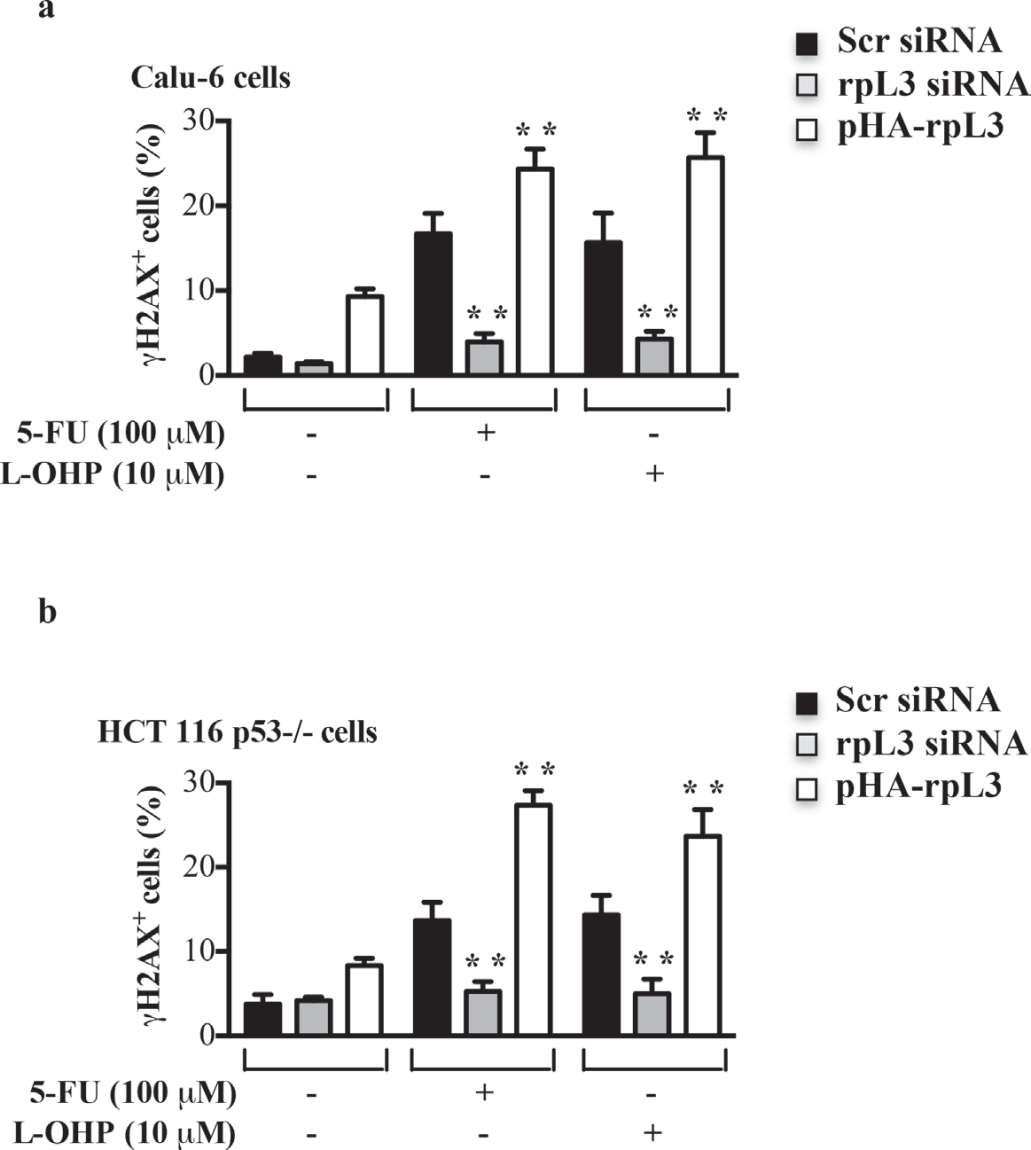
Effect of rpL3 on DNA damage **(a)** Calu-6 cells and **(b)** HCT 116 p53−/− cells were transiently transfected with siRNA specific for rpL3 or scrambled siRNA (Scr) or pHA-rpL3 plasmid. Then, cell were treated with 100 μM 5-FU or 10 μM L-OHP for 24 h or untreated. The samples were immunostained with an anti-γ-H2AX monoclonal antibody followed by secondary fluorescein conjugate antibodies and analyzed by flow cytometry.

In order to analyze whether rpL3 was involved in the DNA damage induction we mesaured the amount of H2AX phosphorylation at serine 139 (termed γ-H2AX), a central component of various signaling pathways in response to DSBs [27]. To this aim, Calu-6 and HCT 116 p53−/− cells were transiently transfected with rpL3 siRNA, scrambled siRNA or pHA-rpL3, a plasmid encoding rpL3-HA. Then, cells were treated with 100 μM of 5-FU or 10 μM of L-OHP for 24 h. Figure 4 showed that in untreated cells the depletion of rpL3 did not cause an increase of DNA damage. In fact, in this condition the levels of γ-H2AX were similar to those observed in untreated cells transfected with scrambled siRNA. In contrast, an increase of γ-H2AX amounts was detected in untreated cells after rpL3 over-expression. As expected, the exposure of cells to 5-FU and L-OHP induced an increase of γ-H2AX levels. The overexpression of rpL3 in these cells caused an additional increase of γ-H2AX indicating that rpL3 is able to increase DNA damage induced by 5-FU or L-OHP treatment. Of interest, the specific silencing of rpL3 abolished DNA damage caused by 5-FU and L-OHP in each cell line (Figure 4).

### rpL3 is involved in HR and NHEJ pathways

**Figure 5:**
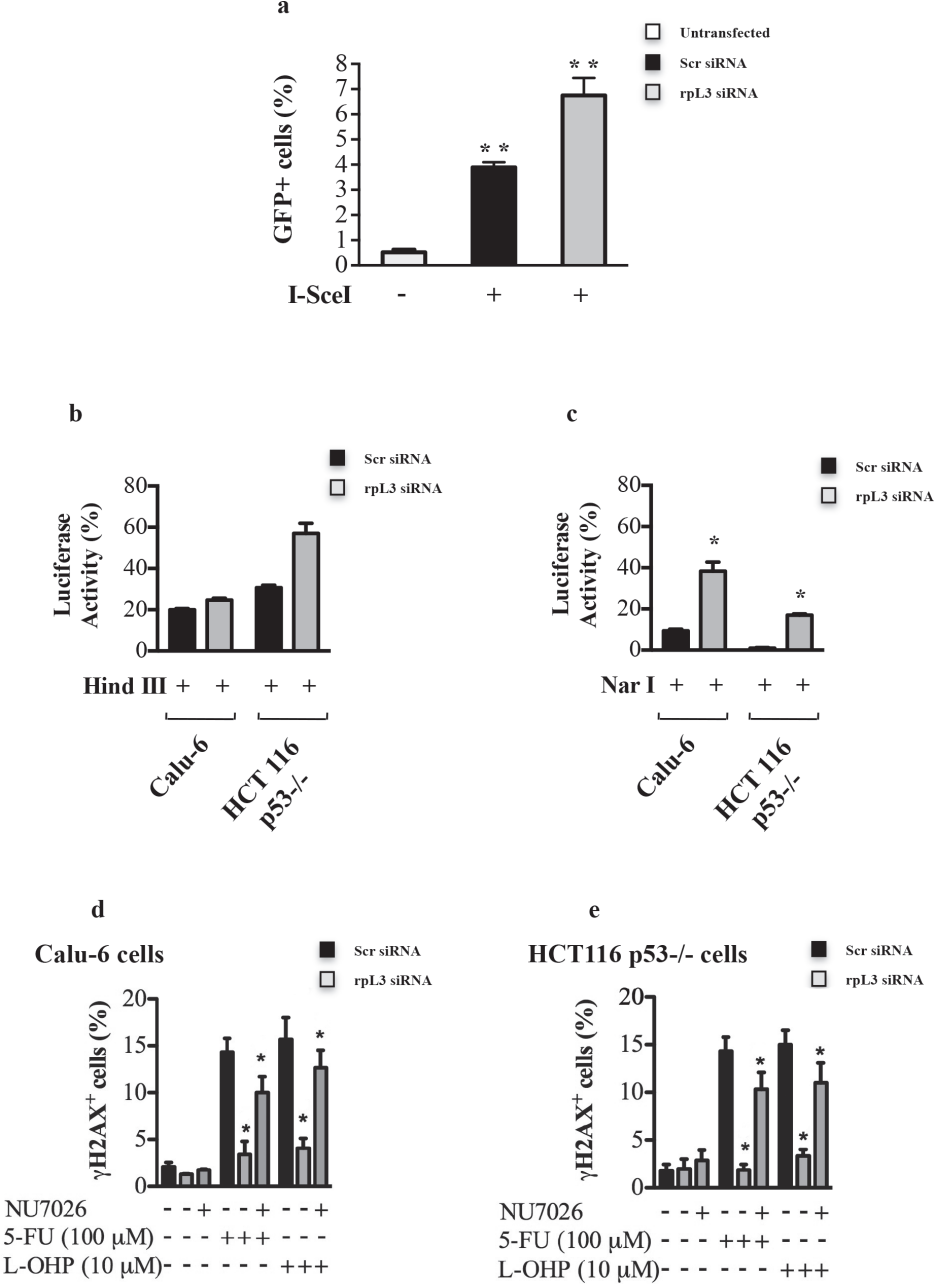
Role of rpL3 in the Homologous Recombination and Non-homologous End Joining **(a)** HeLa/DR-GFP cells were transiently cotransfected with a plasmid expressing the I-SceI enzyme and siRNA specific for rpL3 or scrambled siRNA (Scr). 48 h later, cells were assayed for GFP expression by flow cytometry. Calu-6 cells and HCT 116 p53−/− cells transiently cotransfected with pGL3-luciferase linearized by the restriction endonuclease HindIII **(b)** or NarI **(c)** and siRNA specific for rpL3 or scrambled siRNA (Scr). 48 h later cells were analyzed for the relative luciferase activity, normalized against Renilla Luciferase (pRL). Calu-6 cells **(d)** and HCT 116 p53−/− **(e)** were transiently transfected with siRNA specific for rpL3 or scrambled siRNA (Scr), treated or not with 100 μM 5-FU or 10 μM L-OHP in the presence or in the absence of the DNA-PK inhibitor NU7026. 24 h later, cells were immunostained with an anti-γH2AX monoclonal antibody followed by secondary fluorescein-conjugate antibodies and analyzed by flow cytometry.

In mammals, the formation of DSBs is repaired mainly through two pathways, the homologous recombination (HR) and non homologous end joining (NHEJ) [28, 29]. We assessed the proficiency of cells for HR and NHEJ in conditions of rpL3 silencing. In order to study HR, HeLa/DR-GFP cells (see Materials and Methods) were transiently co-transfected with a plasmid expressing I-SceI endonuclease and rpL3 siRNA or scrambled siRNA (Supplementary Figure 3). 48 h post-transfection, cells were harvested and subjected to GFP flow cytometry. Figure 5a shows that the depletion of rpL3 was associated to an increase of GFP+ cells versus cells transfected with scrambled siRNA.

To examine the involvement of rpL3 in NHEJ, Calu-6 and HCT 116 p53−/− cells were used in a plasmid end-joining assay by using transient transfection of pGL3 plasmid linearized with HindIII or NarI (see Materials and Methods). DNA repair via NHEJ was measured by relative luciferase activity. We observed that depletion of rpL3 affected luciferase production in each cell line (Figures 5b and 5c). Of interest, a significant increase of precise (error-free) DNA EJ activities in cells silenced for rpL3 compared to cells treated with scrambled siRNA was observed (Figure 5c). To better assess the role of rpL3 in the regulation of HR and NHEJ we evaluated the effect of rpL3 on DNA damage after the inhibition of DNA-PK, an essential component of both DNA repair pathways. To this aim, Calu-6 and HCT 116 p53−/− cells were transiently depleted of rpL3 by using specific siRNAs, and DNA-PK was pharmacologically inhibited by using NU7026. Then, cells were treated with 100 μM 5-FU or 10 μM L-OHP and assayed for H2AX phosphorylation by flow cytometry. Figures 5d and 5e showed that after NU7026 treatment, 5-FU or L-OHP treatment of rpL3-depleted cells was associated to a strong increase of phosphorylated H2AX levels. These results suggest that rpL3 is implicated in cell response to drugs treatment by preventing DNA repair via HR and NHEJ.

### rpL3 regulates p21 gene transcription upon 5-FU or L-OHP treatments

We have previously reported that rpL3 is able to control p21 promoter activity [21]. Here, we investigated whether the reported effects of rpL3 in the cell response to drug treatments were related to its ability to modulate p21 expression. To this aim, we evaluated the effect of alterations in rpL3 amount on p21 promoter activity after drug treatments. Calu-6 and HCT 116 p53−/− cells, untreated or treated with 100 μM of 5-FU or 10 μM of L-OHP for 24 h, were collected and subjected to Chromatin immunoprecipitation experiments by using anti-rpL3 antibodies and anti-IgG as control.

**Figure 6:**
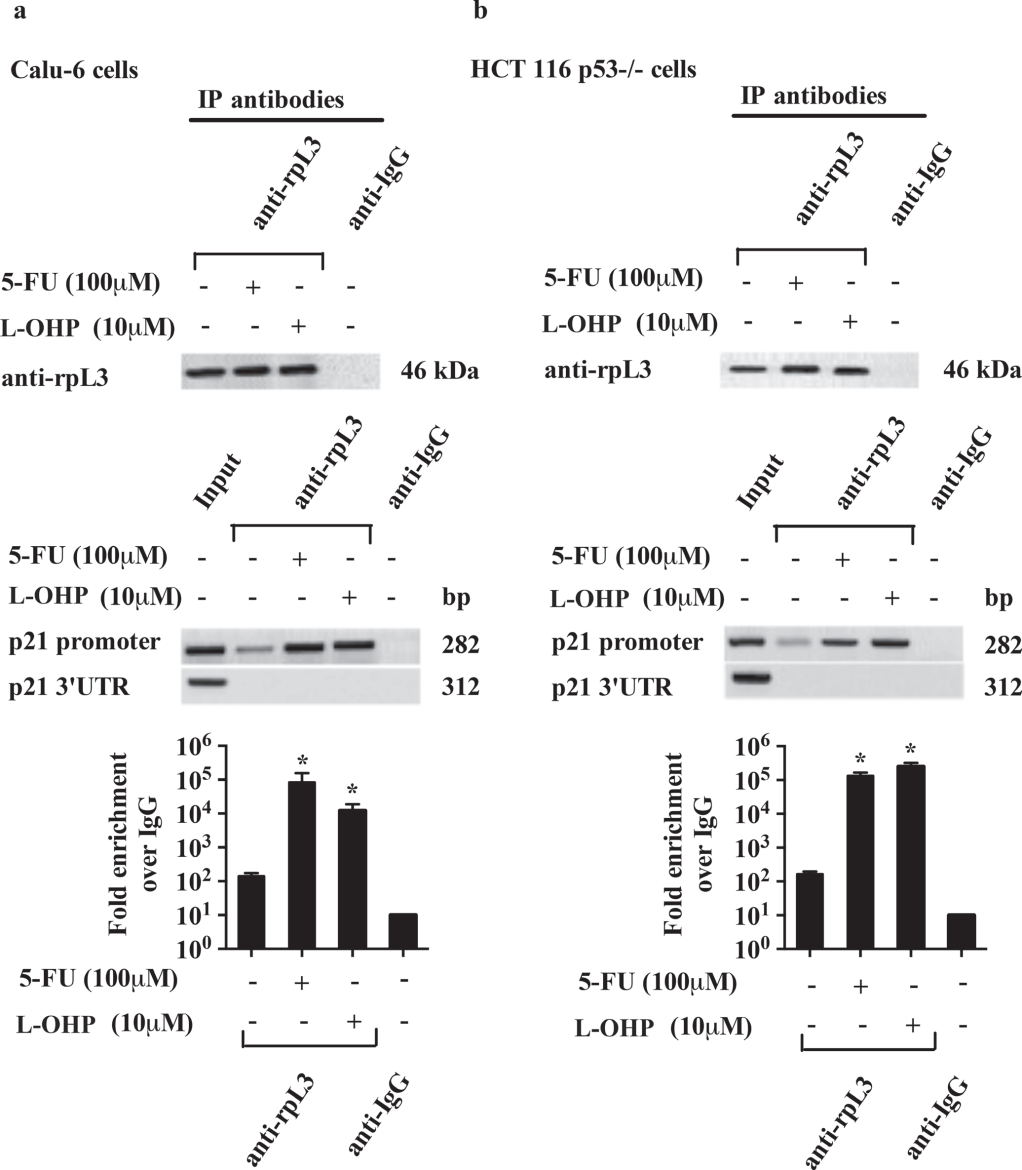
Analysis of the interaction between rpL3 and p21 gene promoter in response to 5-FU and L-OHP treatments Protein samples of DNA-rpL3 or DNA-IgG immunocomplexes from **(a)** Calu-6 cells and **(b)** HCT 116 p53−/− cells untreated or treated with 100 μM 5-FU and 10 μM L-OHP for 24 h were analyzed by western blotting assay with antibodies against rpL3. Note the absence of signal in DNA-IgG immunocomplex. The same DNA-immunoprecipitates were subjected to qPCR with primers specific for the proximal region of p21 gene promoter or control loci (p21 3′UTR).

The presence of rpL3 in DNA-immunoprecipitated complexes was assayed by western blotting. Quantitative PCR (qPCR) assays on the samples were performed as previously reported [21]. Figures 6a and 6b show that in untreated cells, rpL3 is able to bind p21 promoter, as we previously demonstrated [21]. After 5-FU or L-OHP treatment, the binding of rpL3 on p21 promoter was significantly increased compared to that observed in the control.

**Figure 7:**
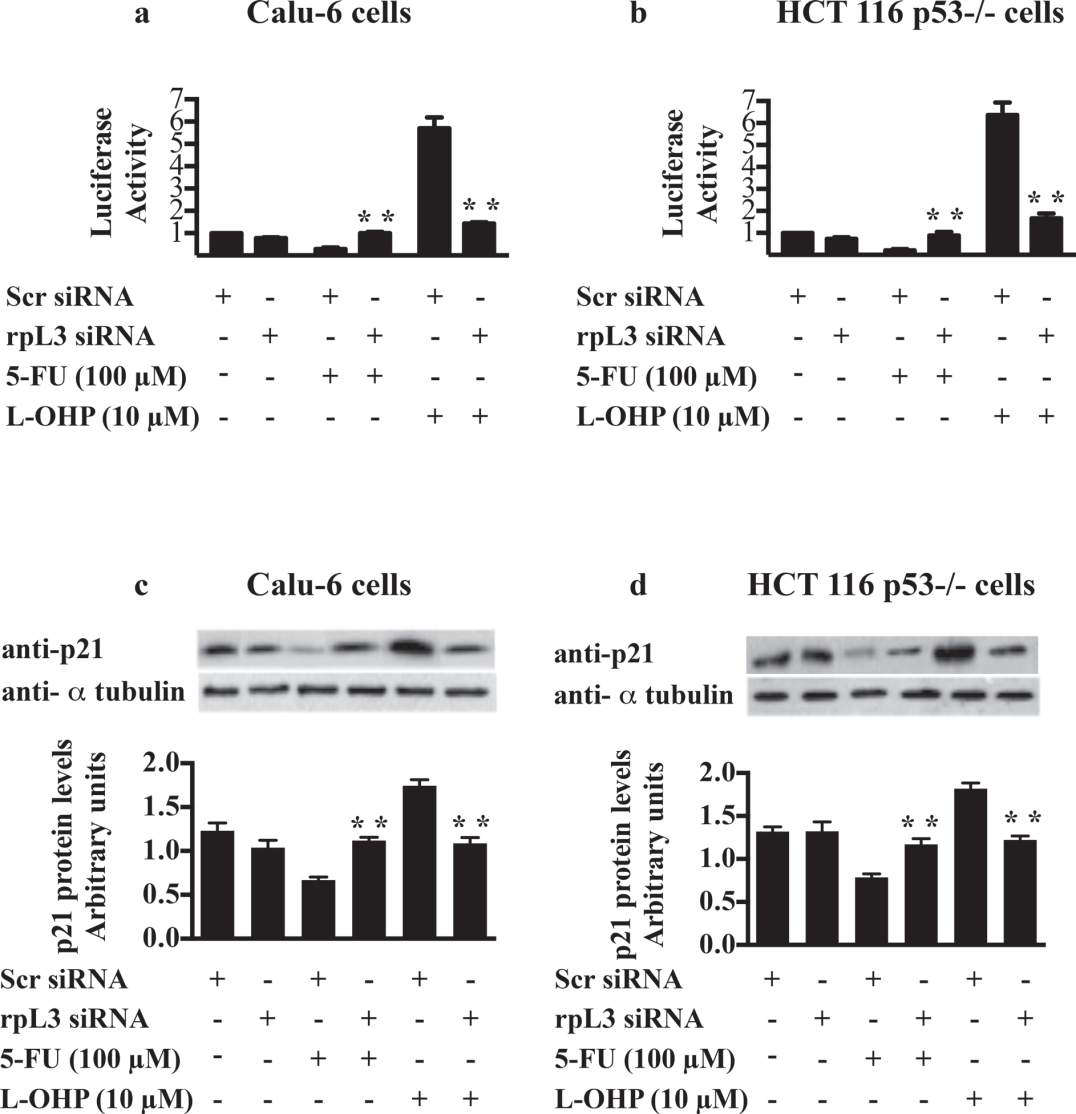
Role of rpL3 in the regulation of p21 promoter activity upon 5-FU and L-OHP treatments **(a)** Calu-6 cells and **(b)** HCT 116 p53−/− cells were transiently cotransfected with the full-length p21 promoter luciferase reporter plasmid and siRNA specific for rpL3 or scrambled siRNA (Scr). Then, cells were treated with 100 μM of 5-FU or with 10 μM L-OHP for 24 h and untreated. Analysis of the relative luciferase activity, normalized against Renilla Luciferase (pRL) activity, of the samples is shown. **(c, d)** Western blotting of protein extracts from the same samples with antibody directed against p21 protein. Loading in the gel lanes was controlled by detection of α-tubulin protein.

Next, to test the role of rpL3 on p21 promoter activity in the response to drug exposure, we performed a reporter luciferase assay in condition of rpL3 silencing and drug treatments. To this aim, Calu-6 and HCT 116 p53−/− cells were transiently cotransfected with the full-length p21 promoter luciferase reporter plasmid [21] and rpL3 siRNA or scrambled siRNA. Then, cells were treated with 100 μM of 5-FU or with 10 μM of L-OHP for 24 h or untreated and analyzed by western blotting with anti-rpL3 and anti- p21. As shown in Figures 7a and 7b in untreated cells the rpL3 silencing did not cause any alteration on p21 promoter activity compared to untreated cells transfected with scrambled siRNA (control). The treatment with 10 μM of L-OHP activated p21 promoter, while the exposure to 100 μM 5-FU reduced p21 gene promoter activity.

When rpL3 expression was switched off, the effect on p21 gene transcription of 100 μM of 5-FU or 10 μM of L-OHP was completely abolished since p21 promoter activity was similar to that observed in the control (Figures 7a and 7b). These data indicated that rpL3 was necessary for regulating p21 promoter activity in the cell response to 5-FU and L-OHP.

We analyzed also the expression levels of p21 protein in these conditions. As shown in Figures 7c and 7d, the treatment with 5-FU caused a reduction of p21 amounts, whereas the addition of L-OHP enriched p21 levels. When rpL3 was silenced, 5-FU and L-OHP failed to exert their effects. In fact, in these conditions p21 levels resulted similar to the basal levels present in the control.

### rpL3 is involved in p21-dependent and p21-independent DNA repair

**Figure 8:**
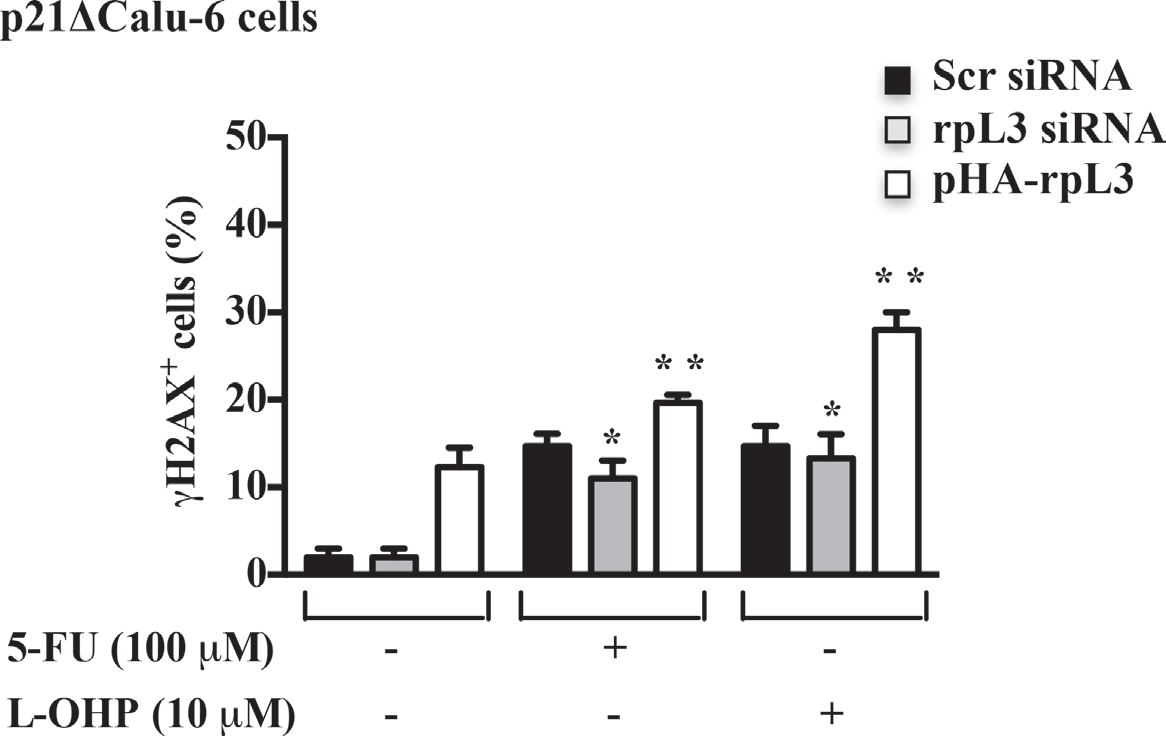
Role of p21 in the rpL3-mediated control of DNA repair p21ΔCalu-6 cells transiently transfected with siRNA specific for rpL3 or scrambled siRNA (Scr) or pHA -rpL3, untreated and treated with 100 μM 5-FU or 10 μM L-OHP for 24 h were immunostained with an anti-γ-H2AX monoclonal antibody followed by secondary FITC-conjugate antibodies and analyzed by flow cytometry.

To understand whether rpL3 was involved in the control of DNA repair through a molecular mechanism mediated by p21, we used p21-depleted Calu-6 (p21ΔCalu-6) cells in which p21 gene expression was stably switched off [21]. We measured DSB formation in p21ΔCalu-6 cells following alterations of rpL3 expression levels. Cells were incubated with rpL3 siRNA or scrambled siRNA or with the plasmid pHA-rpL3. 24 h after transfection, cells were treated with 100 μM of 5-FU and 10 μM of L-OHP. Untreated and treated cells were subjected to γ-H2AX quantification to monitor DSB formation by flow cytometry. Figure 8 shows that in untreated cells, the specific depletion of rpL3 did not affect DSB formation while the ectopic expression of rpL3 induced an increase of DNA damage.

In treated cells, the silencing of rpL3 did not alter DSB levels indicating that p21 was required for the rpL3-mediated control of DNA repair. In fact, in these conditions the levels of γ-H2AX were similar to those observed in treated cells silenced with scrambled siRNA. Furthermore, in treated cells the overexpression of rpL3 was still able to increase drug-induced DNA damage. Taken together, these results indicate that rpL3 is able to control DNA repair in a p21-dependent manner as well as through mechanisms independent from p21 status of cell.

## DISCUSSION

The sensitivity variation and the development of resistance limit the efficiency of chemotherapy [30]. Several preclinical studies have demonstrated that the loss of p53 function reduced cellular sensitivity to 5-FU [9] but the molecular mechanism by which this occurs is still a matter of debate [31]. A better understanding of the molecular mechanism of anti-cancer molecules such as 5-FU and L-OHP, widely used for therapy of a variety of solid tumors [32], may contribute to improve therapy based on these drugs. The results reported here extend the scenario of mechanisms of drugs as 5-FU and L-OHP that specifically impact ribosome biogenesis for the treatment of cancers lacking active p53 and highlight the importance of human rpL3 as critical mediator of cell response to chemotherapy. Data reported here demonstrate that after ribosomal stress induced by 5-FU and L-OHP rpL3 is up-regulated and accumulated as ribosome-free form (Figures 1a and 1b).

5-FU or L-OHP treatments caused a cell cycle arrest and an increase of γ-H2AX levels indicative of a high number of DSBs [33, 34] (Figures 2a and 2b, Figures 4a and 4b). In these conditions the percentage of apoptotic cells was about 20–30% (Figures 3a and 3b) in p53 mutated and p53 null cell lines. The specific rpL3 impairment was able to completely abolish the cytotoxic effects of either 5-FU or L-OHP. In fact, when rpL3 expression was switched off we observed a strong reduction of DNA damage following drug incubation (Figures 4a and 4b) and rpL3-depleted cells not only survived but became able to reenter cell cycle and proliferate (Figure 3c). In addition, the ectopic expression of rpL3 either in untreated cells or treated cells induced γ-H2AX formation (Figures 4a and 4b) strongly suggesting that rpL3 exterts itself cytotoxic effects leading to increase the susceptibility of tumor cells to chemotherapy.

We hypothesized that the absence of citotoxicity in rpL3-depleted cells exposed to 5-FU or L-OHP was due to an activation of DNA repair. Damage to DNA can occur in all cell cycle phases in proliferating cells and repair mechanisms involved vary in the different phases of the cell cycle [35, 36]. Since rpL3 seems to be involved in cell response to drugs that affect different phases of cell cycle, we supposed that rpL3 is able to influence different DNA repair pathways. In fact, our data indicate a strong correlation between intracellular levels of rpL3 and the activity of specific DNA repair processes such as HR and NHEJ implying a significant role for rpL3 in the regulation of DNA repair process (Figure 5). In particular, our results show that rpL3 has a strong effect in inhibiting the precise NHEJ known as classical end joing (Figure 5c).

Next, we verified whether rpL3's effect on DNA repair processes occurred through p21 involvement. Although we observed an increase of rpL3 after treatment with either 5-FU or L-OHP, a different behavior of p21 was observed. In fact, 5-FU caused a decrease of p21 protein level whereas L-OHP was able to induce a marked increase of it (Figures 7c and 7d). Data from ChIP experiments and reporter luciferase assays showed that rpL3 affected p21 expression at transcriptional level acting as transcriptional activator or repressor in response to L-OHP or 5-FU, respectively (Figures 6 and 7). Our previous experiments demonstrated the existence of rpL3-NPM complex involved in the trans-activation of p21 promoter [21]. Since it is known that L-OHP is able to promote the nucleoplasmic translocation of NPM [4] we hypothesize that after L-OHP-induced nucleolar disruption, NPM is released from nucleolus and could be recruited by ribosome-free rpL3 to form rpL3-NPM complex active in p21 promoter induction. In contrast, 5-FU does not induce the translocation of NPM into the nucleoplasm indicating that different protein factors could take part in rpL3-mediated regulation of p21 induced by this drug.

Nowadays the role of p21 in DNA repair has been poorly characterized [37–39]. Our results indicate that in p21-depleted cells, treated with 5-FU or L-OHP, rpL3 silencing did not cause a reduction of DNA damage indicating that p21 was essential for rpL3 role to DNA repair (Figure 8). Of interest, the ectopic expression of rpL3 in untreated and drug-treated p21-depleted cells was associated to an increment of DNA damage suggesting that rpL3 inhibited DNA repair also independently from p21 (Figure 8).

To our knowledge a relationship between r-proteins and inhibition of DNA repair was not described. It is plausible that upon drug-induced ribosomal stress ribosome-free rpL3 could inhibit DNA repair directly or alternatively indirectly by repressing DNA repair proteins as p21. The identification of DNA repair proteins involved as well as rpL3-associated factors could clarify the role of free rpL3 in DNA repair.

The observation that in the absence of chemotherapeutic treatments, rpL3 did not affect p21 promoter transactivation, cell cycle phases, DNA damage content and apoptosis strongly indicate that the extraribosomal function of rpL3 to control cell cycle and/or apoptosis through p21 regulation represents a pathway that is specifically activated upon drug-induced nucleolar stress.

Since reported data demonstrate that intracellular level of rpL3 can deeply influence cell response to drug treatments, the knowledge of rpL3 status in p53 null cancers may have a significant value in terms of the efficacy of chemotherapy based on 5-FU and L-OHP.

In the light of these findings we hypothesize a working model by which the cell response to ribosomal stress caused by 5-FU and L-OHP is strongly influenced by the status of rpL3.

**Figure 9:**
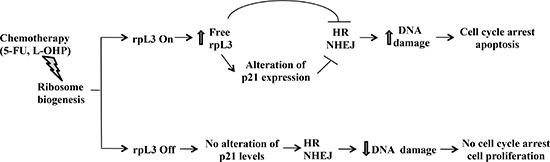
Schematic representation of proposed model In the presence of rpL3 (rpL3 On), drug induced ribosomal stress caused an induction of rpL3 total intracellular levels and the accumulation of rpL3 as ribosome-free form. Free rpL3, directly and/or through alteration of p21 expression, inhibits HR and NHEJ processes leading to an increase of DNA damage and, in turn, to cell cycle arrest and apoptosis. In the absence of rpL3 (rpL3 Off), drug induced ribosomal stress did not associate to cell death. In this condition, no alteration of p21 level, HR and NHEJ were observed, cells survive and proliferate.

We predict that in physiological conditions, rpL3 primarily resides in the ribosome and the intracellular amount of ribosome-free rpL3 is functional to tightly regulate its own expression [40]. Under specific ribosomal stress (i.e. 5-FU or L-OHP treatments), rpL3 could be protected from degradation, as occurred for some r-proteins [24], and accumulates outside the ribosome. The increased ribosome-free rpL3 could translocate from nucleolus to the nucleus where it might directly affect HR and NHEJ or alternatively indirectly by modulating p21 expression levels leading to an increase of DNA damage and, consequently, cell cycle arrest and apoptosis (Figure 9).

The cytotoxic effects of 5-FU and L-OHP including DNA damage, changes in cell cycle and apoptosis, failed to occur after the loss of rpL3 leading to cancer progression (Figure 9).

In conclusion, our results add new insights to the understanding of molecular mechanisms of 5-FU and L-OHP in p53 null tumors. In particular, we demonstrate that human rpL3 acts as stress sensing molecule essential to cell response to 5-FU and L-OHP chemotherapy in cancer lacking active p53.

## MATERIALS AND METHODS

### Cell cultures, transfections and drug treatments

Human cell lines Calu-6 [40], p21ΔCalu-6 [21], HCT 116 p53−/− and HeLa/DR-GFP were cultured in Dulbecco's Modified Eagle's Medium (DMEM) with glutamax (Invitrogen, Carlsbad, California) supplemented with 10% fetal bovine serum (FBS), 2 mM L-glutamine and penicillin-streptomycin 50 U/ml. Calu-6 and p21ΔCalu-6 cell medium was also supplemented with 0.1 mM non-essential amino acids (Euroclone, West York, UK).

siRNA and plasmid transfections were performed in cells as previously described [20, 21].

The siRNA targeting rpL3 was purchased from Santa Cruz Biotechnology (Santa Cruz Biotechnology, Santa Cruz, CA, USA, sc-76400).

Drug treatments were performed after siRNA or DNA transfection by adding to cells 100 μM of the 5-FU, 10 μM of the L-OHP or 5 nM of ActD (Sigma-Aldrich, St. Louis, MO, USA) for 24 h. To block DNA-PK cells were treated with 10 μM of NU7026.

### Ribosome isolation

Calu-6 or HCT 116 p53−/− cells were pelleted and resuspended in lysis buffer (10 mM NaCl, 10 mM MgCl2, 10 mM Tris–HCl (pH 7.5), 0.5% NP-40, aprotinin 1 mg/ml, leupeptin 1 mg/ml, pepstatin A 1 mg/ml, phenylmethylsulfonyl fluoride 100 mg/ml). After incubation in ice for 10 min, the extract was centrifuged for 1 min in a microcentrifuge at a maximum speed at 4°C and the supernatant (total extract) was frozen in liquid nitrogen. To isolate ribosomes and ribosomal subunits, the total extracts were spun at 100 000 g for 2 h on 15% sucrose cushion. After centrifugation, the pellet (which includes polysomes and ribosomal subunits) was resuspended in Loading Buffer (63 mM Tris–HCl pH 6.8, 5% glycerol, 1% SDS, 2.5% bromophenol-blue) for western blot analysis, whereas the supernatant was precipitated with 10% trichloroacetic acid and resuspended in Loading Buffer for western blot analysis.

### Western blotting

Western blotting analysis was performed as previously reported [41]. The membranes were challenged with anti-rpL3, anti-rpL7a (Primm, Milan, Italy), anti-rpS19 (Sigma-Aldrich), anti-p21 (Sigma-Aldrich), and anti-α-tubulin (Santa Cruz Biotechnology). Proteins were visualized with enhanced chemiluminescence detection reagent according to the manufacturer's instructions (Pierce, Rockford, Illinois).

### Chromatin immunoprecipitation

Chromatin immunoprecipitation assay was performed as previously reported [21].

### Dual luciferase assay

Luciferase assays were performed with the Dual-Glo Luciferase assay system (Promega, Madison, Wisconsin, USA) following manufacturer's instructions. Samples were read with Turner Luminometer and expressed as relative luciferase, i.e., RT/RC, where RT and RC are (Firefly luciferase)/(Renilla luciferase).

### BrdU incorporation

For BrdU incorporation, Calu-6 cells or HCT 116 p53−/− cells were labeled for 40 min with 20 μM BrdU (Sigma-Aldrich), harvested and fixed in 70% ethanol. Subsequently, cells were incubated with 20 μl of anti-BrdU-FITC antibody (BD Biosciences, Italy) for 45 min in dark at room temperature. Cells were then washed twice with PBS 1 × and Tween 0.1%, centrifuged and resuspended in 1 ml of PBS containing 10 μg/ml of RNase and 5 μg/ml of propidium iodide (Sigma-Aldrich). Samples were analyzed by a CyAn ADP Flow Cytometer (DAKOCytomation) and quantified using Summit Software.

### Mitochondrial membrane potential measurement

To quantify changes in mitochondrial membrane potential, Calu-6 cells or HCT 116 p53−/− cells were labeled with 50 nM of the mitochondrial membrane potential–sensitive fluorescent dye TMRE (Invitrogen), for 30 min at 37°C, analyzed by a CyAn ADP Flow Cytometer (DAKOCytomation) and quantified using Summit Software.

### Flow cytometry for γ-H2AX

To detect γ-H2AX, cells were fixed with 70% ethanol in PBS and routinely kept at −20°C overnight. Cells were washed with PBS and permeabilized with PBS, 4% fetal bovine serum, and 0.1% Triton X-100 for 10 min on ice. Cells were incubated with anti-γ-H2AX monoclonal antibody (JBW301 from Upstate Biotechnology), in a 1:200 dilution in PBS, 4% fetal bovine serum, for 2 h. Cells were washed twice with PBS-T (PBS, Tween-20 0.1%) and incubated with 1:200 dilution of fluorescein-tagged goat anti-mouse secondary antibody (Santa Cruz Biotechnology).

After washes with PBS-T, cells were resuspended in PBS and were analyzed using a CyAn ADP Flow Cytometer (DAKOCytomation) and Summit Software.

### Clonogenic assays

For clonogenic assay, Calu-6 cells cells were plated in triplicate at 4 × 10^3^ in 6-well multidishes. After 5 to 7 days, colonies were stained with 1% methylene blue in 50% ethanol.

### *In vivo* recombination assay system and analysis of homologous recombination frequency

HeLa/DR-GFP cells carrying a single integrated copy of GFP recombination reporter construct were kindly provided by Prof. E. Avvedimento. This reporter construct contains two GFP genes: one nonfunctional GFP gene mutated to contain a I-SceI cleavage site and an additional, truncated GFP gene that can correct the SceI site mutation. The I-SceI restriction endonuclease is used to introduce a DSB in the reporter gene. Chromosomal repair of the reporter gene by homologous recombination leads to GFP expression, which is analyzed by flow cytometry.

To examine the effect of rpL3 on DSB-induced homologous recombination, HeLa cells were transiently transfected with the I-SceI expression vector pCAGGS-ISceI, in the presence or in the absence of siRNA for rpL3 or scrambled siRNA. 48 h later, the percentage of GFP-positive cells was determined by flow cytometry using a CyAn ADP Flow Cytometer (Beckman Coulter, Inc., Milano, Italy) and Summit Software.

### *In vivo* end-joining assay

Plasmid pGL3 basic (Promega) was linearized with HindIII (recognition site between the promoter and the luciferase cDNA) or NarI (cleaves within the coding region of the luciferase cDNA). Cells were transiently cotransfected with circular, linearized plasmid and pRL-SV40 Renilla luciferase plasmid, in the presence or in the absence of rpL3 siRNA or scrambled siRNA. After 48 h, cells were lysed in passive lysis buffer and analyzed by the dual luciferase assay (Promega). Repair efficiency was calculated from the luciferase activities of linearized reporter constructs compared with that of the intact plasmid.

### Statistical analysis

Error bars represent mean ±s.d. from *n* = 3 biological replicates. *P < 0.05 was considered significant, **P < 0.01 was considered highly significant; Student t-test is used throughout.

## SUPPLEMENTARY FIGURES


