# Supplementary material for: Human rpL3 plays a crucial role in cell response to nucleolar stress induced by 5-FU and L-OHP
Source: Oncotarget. 2014 Nov 28;5(22):11737–51. doi: 10.18632/oncotarget.2591 (PMC4294388; doi:10.18632/oncotarget.2591)
Supplement: Supplementary file 1 [file oncotarget-05-11737-s001.pdf]

SUPPLEMENTARY FIGURES

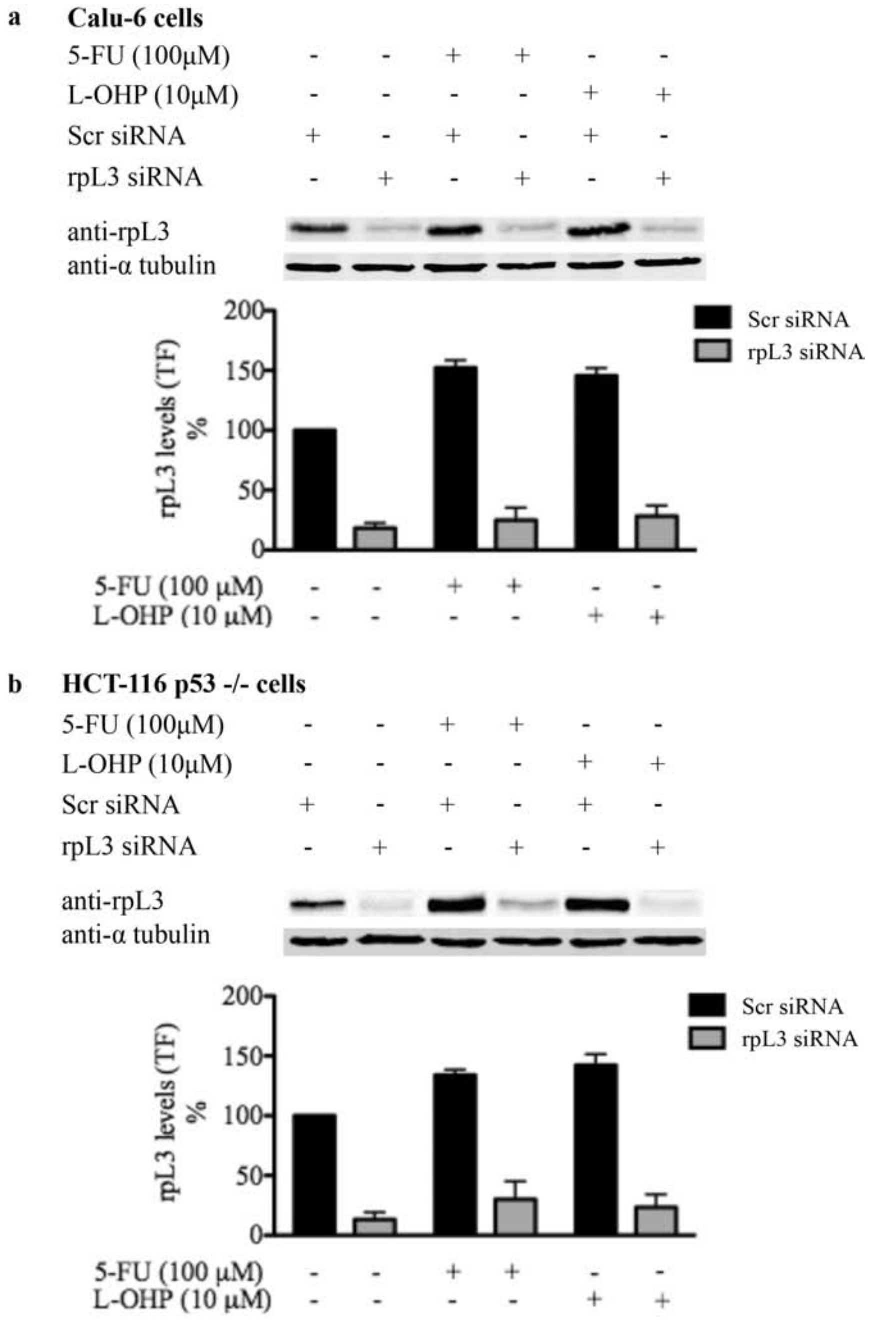

Supplementary Figure S1: rpL3 silencing in Calu-6 cells and HCT 116 p53-/- cells. Protein samples from **(a)** Calu-6 cells and **(b)** HCT 116 p53-/- transiently transfected with siRNA specific for rpL3 or scrambled siRNA (Scr), and treated with 100 µM 5-FU or 10 µM L-OHP for 24 h or untreated were analyzed by western blotting assay with antibodies directed against rpL3. Loading in the gel lanes was controlled by detection of α-tubulin protein. Quantification of rpL3 in TF (total fraction) is shown. Results illustrated in Figure S1 are representative of all experiments in which rpL3 was transiently silenced.

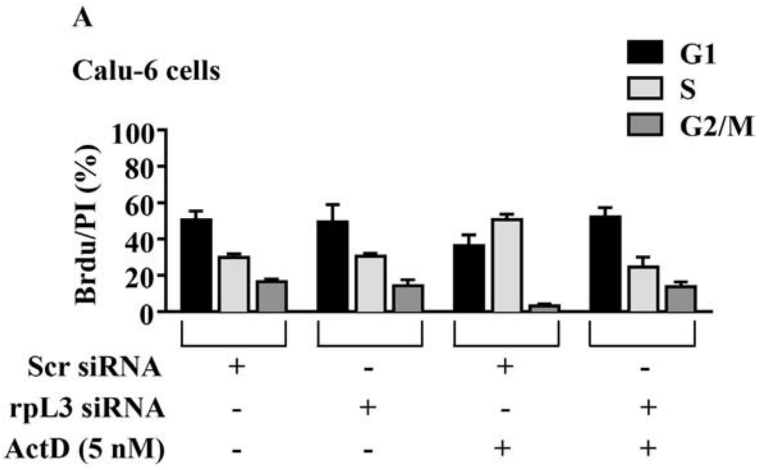

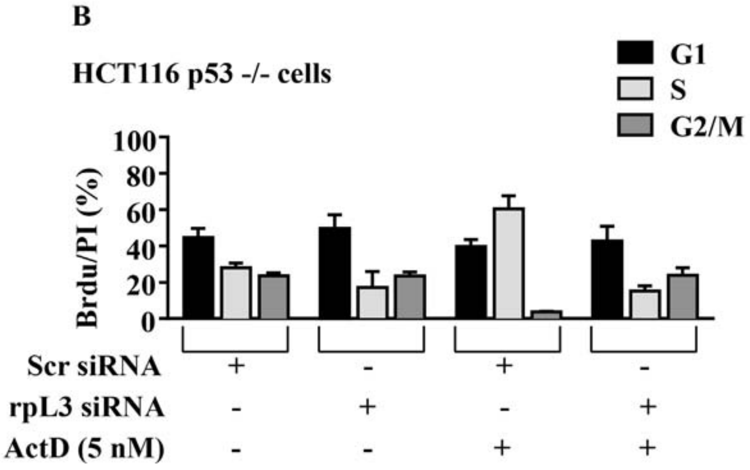

Supplementary Figure S2: Calu-6 cells and HCT 116 p53-/- cells were transiently transfected with siRNA specific for rpL3 or scrambled siRNA (Scr), and treated with 5 nM ActD for 24 h or untreated. Cells were stained with FITC conjugated anti-5-bromodeoxyuridine antibodies and counterstained with propidium iodide to analyze DNA synthesis and DNA content respectively by flow cytometry. The percentage of cells in different phases of cell cycle is shown.

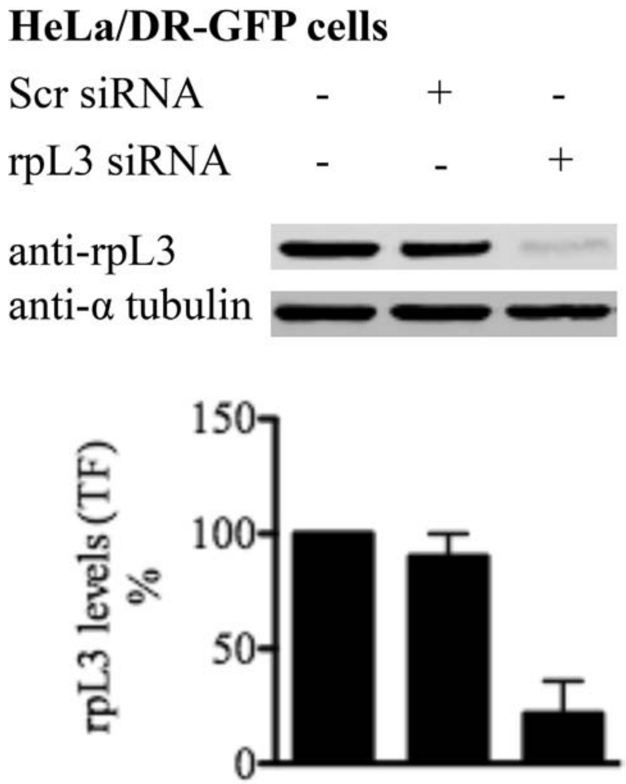

Supplementary Figure S3: rpL3 silencing in HeLa/DR-GFP cells. Protein samples from HeLa/DR-GFP cells transiently transfected with siRNA specific for rpL3 or scrambled siRNA (Scr) or untrasfected were analyzed by western blotting with antibodies directed against rpL3. Loading in the gel lanes was controlled by detection of α-tubulin protein. Quantification of rpL3 in TF (total fraction) is shown.
